# Review on Strategies and Technologies for Exosome Isolation and Purification

**DOI:** 10.3389/fbioe.2021.811971

**Published:** 2022-01-05

**Authors:** Jiaci Chen, Peilong Li, Taiyi Zhang, Zhipeng Xu, Xiaowen Huang, Ruiming Wang, Lutao Du

**Affiliations:** ^1^ State Key Laboratory of Biobased Material and Green Papermaking, Department of Bioengineering, Qilu University of Technology (Shandong Academy of Sciences), Jinan, China; ^2^ Department of Clinical Laboratory, The Second Hospital, Cheeloo College of Medicine, Shandong University, Jinan, China; ^3^ School of Cellular and Molecular Medicine, University of Bristol, Bristol, United Kingdom

**Keywords:** exosomes, microfluidics, exosome isolation, exosome separation, cancer

## Abstract

Exosomes, a nano-sized subtype of extracellular vesicles secreted from almost all living cells, are capable of transferring cell-specific constituents of the source cell to the recipient cell. Cumulative evidence has revealed exosomes play an irreplaceable role in prognostic, diagnostic, and even therapeutic aspects. A method that can efficiently provide intact and pure exosomes samples is the first step to both exosome-based liquid biopsies and therapeutics. Unfortunately, common exosomal separation techniques suffer from operation complexity, time consumption, large sample volumes and low purity, posing significant challenges for exosomal downstream analysis. Efficient, simple, and affordable methods to isolate exosomes are crucial to carrying out relevant researches. In the last decade, emerging technologies, especially microfluidic chips, have proposed superior strategies for exosome isolation and exhibited fascinating performances. While many excellent reviews have overviewed various methods, a compressive review including updated/improved methods for exosomal isolation is indispensable. Herein, we first overview exosomal properties, biogenesis, contents, and functions. Then, we briefly outline the conventional technologies and discuss the challenges of clinical applications of these technologies. Finally, we review emerging exosomal isolation strategies and large-scale GMP production of engineered exosomes to open up future perspectives of next-generation Exo-devices for cancer diagnosis and treatment.

## Introduction

The development of medical technologies has reached an unprecedented level in the 21st century. However, early diagnosis and complete recovery of malignant tumors are still facing severe challenges. Cancers have become a dominating health concern around the world. The rapid surge of cancer morbidity and mortality worldwide has shown more than 19.3 million new cancer cases and an estimated 10.0 million deaths in 2020. With an expected 28.4 million cases in 2040, it is an increase of about 47% from 2020 (Sung et al., 2021). The situation deeply dues to the concealment of cancer and the limitations of invasive tests (e.g., Tissue biopsy) and imaging tests (e.g., CT, Type-B ultrasonic) (Nasrollahpour et al., 2021). Once cancers are diagnosed by the above methods, they often have spread and are in the late stage (Chambers et al., 2002; Nasrollahpour et al., 2021). If the diagnosis can be made in the early stage, patients have a great chance of recovery (Chambers et al., 2002; Chen et al., 2020). For the patients, early diagnosis of cancer not only reduces costs, improves therapeutic effect but is key to reducing mortality (Qian et al., 2019; Whitaker, 2020). Accordingly, early cancer detection plays a significant role and has been a subject of cancer prevention and care.

In the few decades, the emerging liquid biopsies have shown powerful potential as non-invasive diagnostic methods for early cancer detection. Liquid biopsies are the sampling and analysis from various biological fluids of patients to obtain disease-related information, mainly including circulating tumor cells (CTCs), exosomes, and other vesicles (Huang et al., 2021). Among them, exosomes possess valuable information about physiology and pathology.

Exosomes are secreted from almost all cell types and abundantly present in biofluids (Raimondo et al., 2011). Lipid bilayer membranes of exosomes enable them to firmly carry and transmit important biological signals from their cells of origin, which not only affect the physiological state of the body but also are closely related to cell communication (Raimondo et al., 2011), immunoregulation (Huang et al., 2017), angiogenesis (Zhuang et al., 2012), tumorigenesis, and metastasis (Melo et al., 2015; Thind and Wilson, 2016; Zheng et al., 2018; Correa et al., 2020). Thus, exosomes have become promising tools for monitoring cancer occurrence and progression. Despite their potential, our understanding of exosomal functions remains limited. One of the main challenges for research stagnation is the lack of an efficient standardized isolation strategy for specific exosome subpopulations due to their inherent heterogeneity. In addition to the need to separate intact and pure exosomes, exosome isolation approaches will further progress towards high purity, high throughput, low operation time, and repeatability ( Mathivanan et al., 2010a; Yang and Robbins, 2011; An et al., 2019 ).

At present, common exosome isolation technologies, such as ultrafiltration, immunoaffinity, ultracentrifugation (“gold standard” for the isolation of exosomes) are expensive instruments, large volumes of sample, possible protein contamination, complete isolation steps, but they result in low isolation efficiency, sample loss, low exosome recovery and purity (LeBleu and Kalluri, 2020). Advances in nanotechnologies and microfluidics have led to incorporating microfluidics into exosome isolation. Integrated and optimized microfluidic chips will be expected to be promising tools for future research. Although there have been various reviews summarizing the topic of exosome isolation and purification, most of them rarely focused on the advantages and drawbacks of each technology in detail. Moreover, reviews including more updated and promising methods are crucial to timely learn about the latest research contents and trends in this field.

In this review, to better understand the importance and significance of cancer-derived exosomes in early detection and treatment, we first summarized several crucial properties of exosomes. Then, we introduced the conventional isolation methods, such as ultracentrifugation, ultrafiltration, precipitation. Finally, the current pivotal advances in microfluidics and other emerging methods for exosomal purification were presented. To maximize comprehensiveness and visualization, properties of the techniques and strategies catalogued in the review are summarized and organized into two tables (Tables 1, 2).

**TABLE 1:** Comparison of common exosomes isolation methods and their benefits/disadvantages.

Strategy	Principle	Benefits	Disadvantages	Time	Purity	Yield	References
Ultracentrifugation	Components with imparity of size and density possess various sediment speed	Gold standard, suitable for large-volume samples, relatively cheap, mature	Time-consuming, cumbersome operation, low yield, may damage exosomes	> 4 h	Medium (with the co-precipitation and non-exosome contaminants)	Low	Lin et al. (2020)
Density gradient centrifugation	Components with imparity of size and density possess various sediment speed	High purity, avoiding exosomal damage	Labor-intensive, preliminary preparation and cumbersome operation	> 16 h	High	Low	Kamerkar et al. (2017)
Ultrafiltration	Particles with various size and molecular weight	East, without special equipment and reagents	Clogging on filtering membrane, loss of exosomes of small particle diameter	Generally < 4 h	High	Medium	Ding et al. (2021)
SEC	Particles with various size and molecular weight	Simple, economical, maintain the biological function and structure	Special columns and packing are required, lipoprotein contamination	0.3 h for qEV (Izon Science, New Zealand)	High	High	Mohammadi et al. (2021)
Immunoaffinity	Based on interaction between antibodies and specific membrane proteins of exosomes	High specificity for exosome subtypes isolation	Expensive, depending on specificity of the antibody	4–20 h	High	Medium	Coumans et al. (2017)
Polymer precipitation	The influence of exosomal the solubility or dispersibility under the high hydrophilic polymers	Simple operation, suitable for large-volume samples	Potential contaminants (co-purifying protein aggregates or residuary polymers)	≈0.3–12 h	Low	High	Coumans et al. (2017)

**TABLE 2:** Comparison of microfluidics and other emerging approaches for exosome isolation.

Mechanism	Principle	Sample	Working volume	Time	Separation recovery	References
TiO_2_-based exosome isolation process	Interaction between TiO_2_ particles and the phosphate groups on the surface of exosomal lipid membranes	Human serum samples	20 ml	≈5 min	93.4%	Gao et al. (2019)
Fe_3_O_4_@TiO_2_-CD63 aptamer	Double interaction of CD63 DNA aptamer and TiO_2_ for exosomes	Urine samples	≈100 ml	≈10 min	92.6%	Zhang et al. (2021)
ExoCAS-2	Based on exosomal negative charges, polycationic polymers can adhere to exosomes	Plasma samples	1 ml	≈40 min	NA	Kim and Shin, (2021)
Microvortex chip	Nanoprobes can inserted into exosomal lipid bilayer membrane	Cell culture and human serum samples	1 ml	10 h	≈70%	Han et al. (2020)
Acoustofluidic platform	Integration of acoustics and microfluidics	Undiluted human whole blood	100 μL	≈25 min	98.4%	Wu et al. (2017)
Acoustofluidic centrifuge system	Double interaction of droplet spinning and acoustic streaming	Exosome samples	Nanoliters to microliters	≤1 min	80–86%	Gu et al. (2021)
Paper-based anionic ITP device	isotachophoresis	Human serum samples	5 μl	≤10 min	NA	Guo et al. (2020)
Microfluidic nanowire array	Filtration and immunoaffinity	Human breast cells	1 ml	≈20 min	≈70%	Suwatthanarak et al. (2021)
ExoDFF	Based on equilibrium of Dean drag forces and inertial lift	Whole blood	5 ml	< 1 h	≈15%	Tay et al. (2021)
Raman assay chip	Immunomagnetic	Cell culture and serum samples	20 μl	< 1 h	72.5%	Wang et al. (2020)
Lipid microarray	Membrane fusion and immunoaffinity	Cell culture and serum samples	50 μl	≈1 h	NA	Liu et al. (2021a)
EV-CLUE chip	Immunoaffinity	Cell culture and serum samples	5–10 μl	≈1 h	≈78.2% for SKOV3	Zhang et al. (2020)

## Exosomes

**FIGURE 1:**
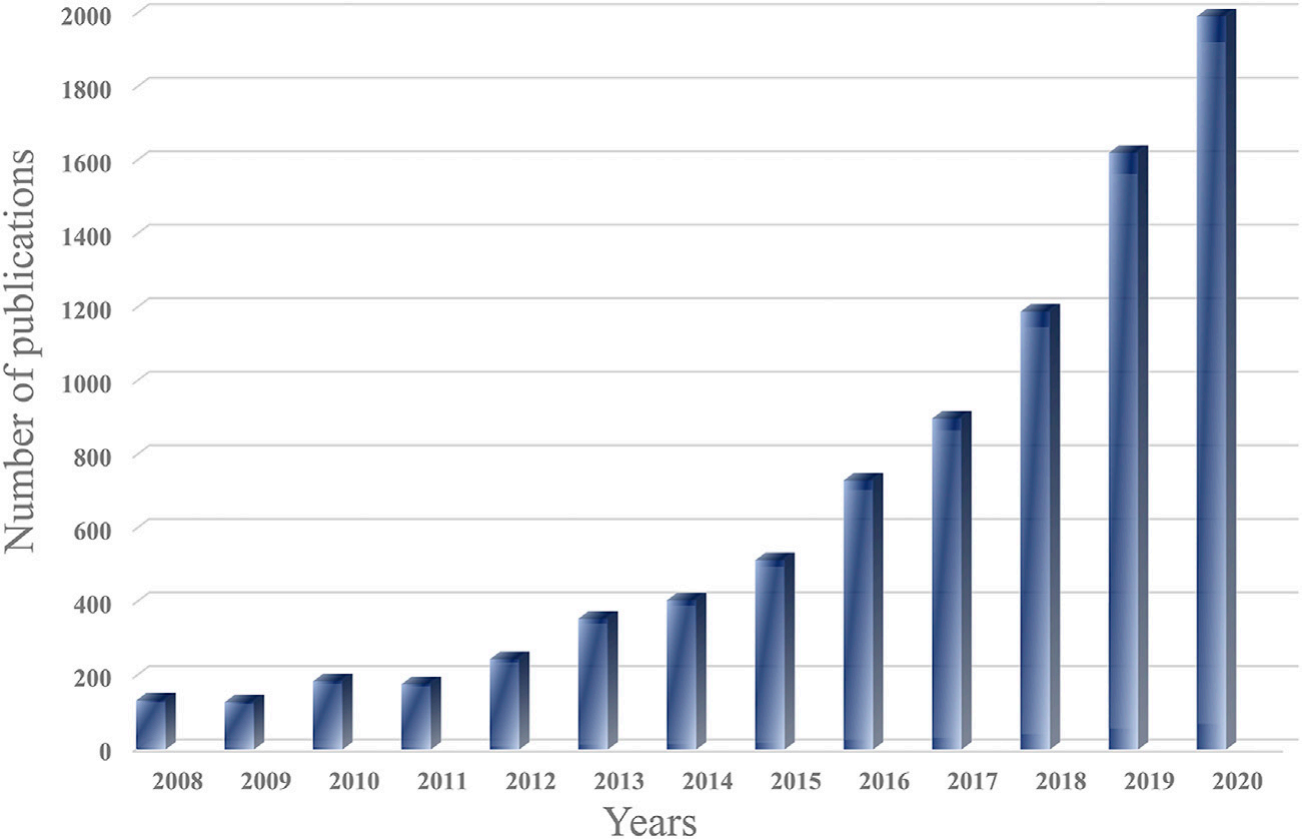
The number of exosomal publications. The graph was generated from Web of Science.

Extracellular vesicles are lipid bilayer-closed structures derived from endocytosis and secreted by almost all types of cells, including exosomes (30–150 nm), microvesicles (150 nm to 1 μm), and apoptotic bodies (1–5 μm) (Murk et al., 2003; Yang and Robbins, 2011 ). For a long time, these vesicles were thought to be a way to load cellular metabolic waste, which is responsible for transporting waste generated by cells (Johnstone et al., 1987). Until 1983, the role of some vesicles with 30–150 nm had been preliminarily identified and named exosomes by Pan and Johnstone when they studied the development of reticulocytes in sheep (Pan and Johnstone, 1983; Johnstone et al., 1987 ). Observed by an electron microscope, the shape of exosomes is cup-shaped or spherical (typically 30–150 nm in diameter) (Zhang et al., 2019). At present, various researches have been conducted on exosomes and showed exosomes play an irreplaceable role in cancer metastasis and normal physiology (Gurwitz, 2015; Zhang et al., 2015; Mateescu et al., 2017; Zhao et al., 2018). Thus, the field of exosomes has been developed unprecedentedly, and the number of related papers has grown exponentially in the last decade (Figure 1).

### Exosomal Biogenesis

**FIGURE 2:**
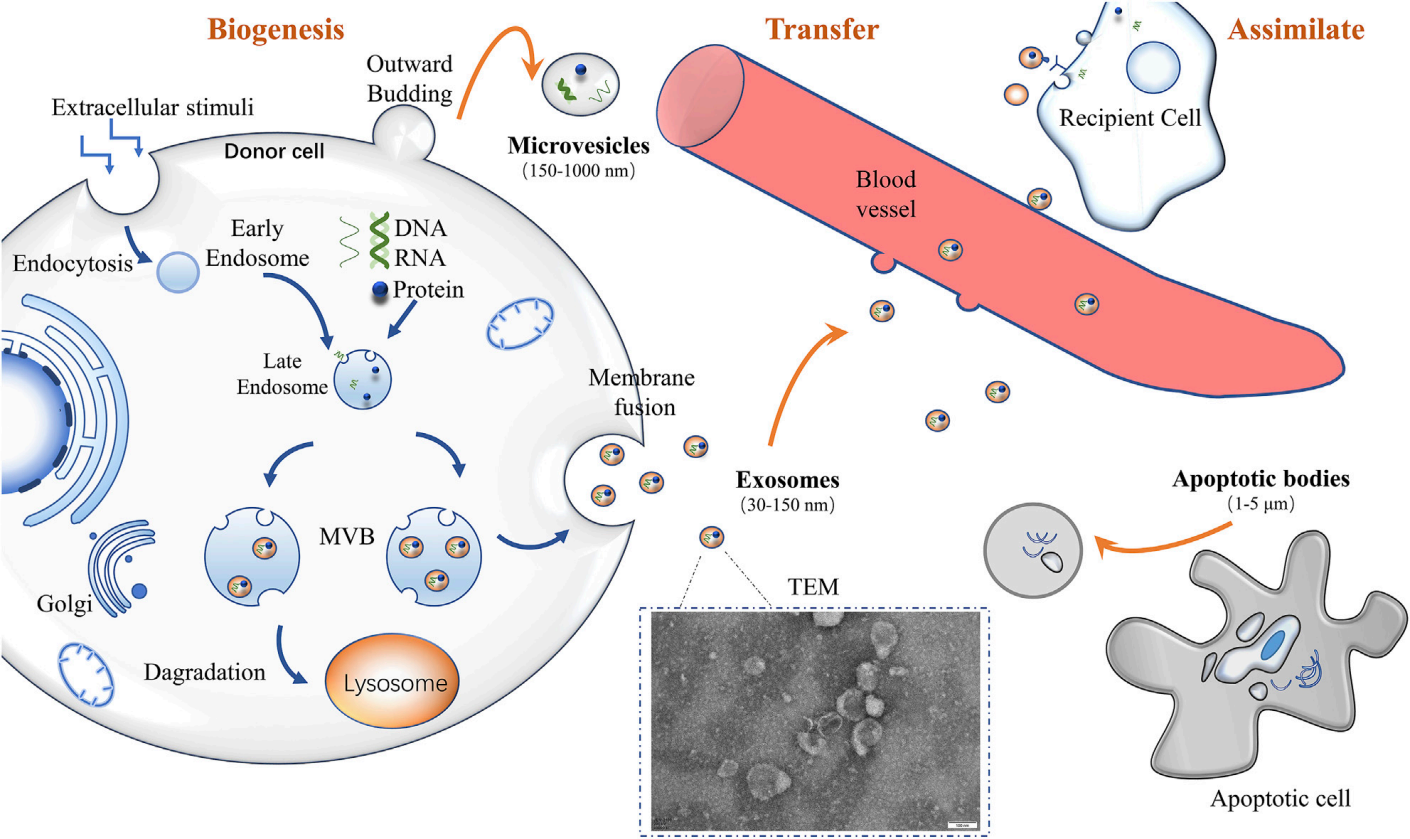
Biogenesis of exosomes and other vesicles (Hessvik and Llorente, 2018) (van der Pol et al., 2012) (Gurunathan et al., 2019).

The term “exosome” was invented to describe a subtype of extracellular vesicles secreted through multivesicular bodies (MVBs) in the endosomal pathway (Raposo and Stoorvogel, 2013 ; Lee et al., 2012). Other subtypes contain apoptotic bodies produced by cells in apoptotic conditions, and microvesicles (MVs) derived from the outward budding of the plasma membrane as shown in Figure 2 ( Lin et al., 2020 ). Compared with each other, these three subtypes differ in size, function, biological origin, and other aspects (Abhange et al., 2021). Exosomes can be induced by many factors, such as microbial and extracellular stimulation or other stress conditions (De Toro et al., 2015). Exosomal biogenesis begins with early endosomes (Figure 2; Lin et al., 2020). Under the influence of a certain factor, the cell membrane undergoes endocytosis to sag inward. The inner membrane formed by this process is called the early endosome (Lee et al., 2012). The early endosomes will continue to migrate within the cell (from the periphery of the cell to the nucleus) and gradually mature (from the original tube to the sphere) (Théry et al., 2002; Keller et al., 2006). When early endosomes mature into late endosomes, the inner body membrane will further develop multiple depressions, encapsulating parts of the cytoplasm and some substances (e.g., nucleic acids, proteins from Golgi and cell nucleus), thereby generating intraluminal vesicles (ILVs) (Keller et al., 2006; Théry, 2011; Clark et al., 2015; Kalluri and LeBleu, 2020). Next, MVBs, the late endosomes with ILVs, can fuse with either the lysosome or the cell membrane (Colombo et al., 2014). Scholars reported cholesterol content on MVBs is closely related to the regulation of this process (Möbius et al., 2002). If MVBs fuse with lysosomes, they will be degraded entirely (Théry et al., 2002; Colombo et al., 2014). Other MVBs fuse with the cell membrane, where their contents (ILVs) could be released into the extracellular environment. The released vesicles are designated as exosomes (Hessvik and Llorente, 2018). These exosomes can interact with the extracellular matrix to affect surrounding cells and transport their contents to target cells through body fluids (blood, urine) (Bebelman et al., 2018; Bebelman et al., 2018). ESCRT (endosomal sorting complex required for transport), Alix, and other related proteins or auxiliary factors play an irreplaceable role in the formation of exosomes (Baietti et al., 2012; Colombo et al., 2013). In addition, some lipids and proteins also regulate the process (Baietti et al., 2012; Pocsfalvi et al., 2016).

### Contents and Functions

**FIGURE 3:**
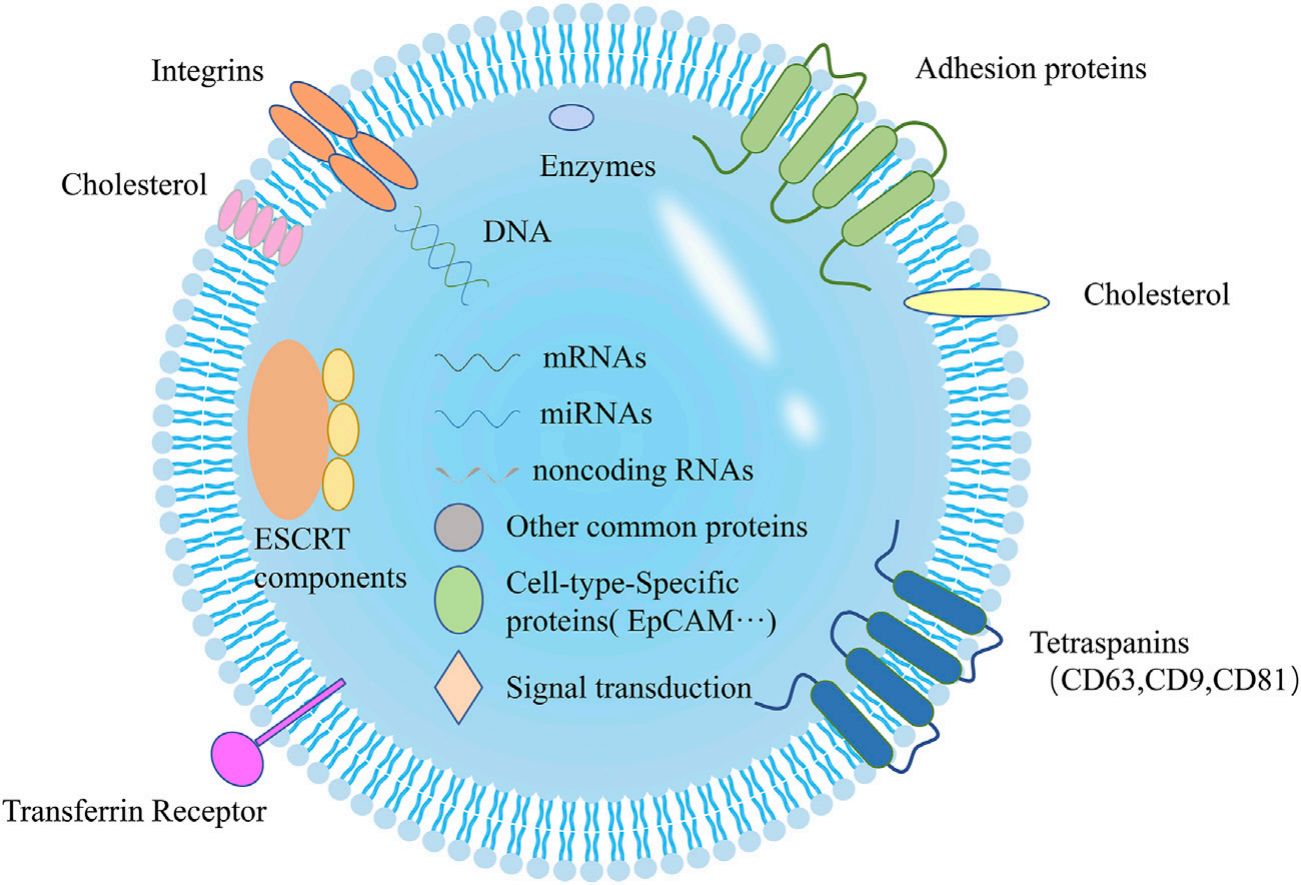
Schematic of exosomal molecular composition. Exosomes contain various important biomarkers, such as proteins, lipids, and miRNAs.

Generally, the exosomal components are unequal depending on their source cell (Figure 3). Until now, studies have shown that nearly 350,000 proteins, 40,000 nucleic acids, and 600 lipids exist in various exosomes (according to http://www.microvesicles.org/). This provides unlimited possibilities for practical clinical scenarios of exosomal disease diagnosis and treatment.

Proteins are considerable components of exosomes, which come from endocytosis, membrane fusion, and cytoplasm of the cell of origin (Zaborowski et al., 2015). However, this does not mean exosomal proteins are random. For instance, the proteins are hardly derived from mitochondria, Golgi apparatus, nucleus, and endoplasmic reticulum (Console et al., 2019). Generally speaking, the proteins can be divided into common and specific proteins. The common proteins exist in almost all exosomes, such as related to membrane transport and fusion (Rab, GTPases, flotillin), synthesis of multivesicular bodies (Alix, TSG 101), tetraspanins (CD9, CD63, CD81, CD82), and cytoskeleton proteins (heat shock protein, actin, tubulin) (Urbanelli et al., 2013; Rajagopal and Harikumar, 2018; Lin et al., 2020). Thus, the presence of exosomes can be confirmed by detecting their common proteins (Shao et al., 2018). The specific proteins derive from their cells of origin. For example, exosomes from malignant ascites secreted by ovarian cancer patients contain epithelial cell adhesion proteins (CD24 and EpCAM) (Runz et al., 2007). Therefore, by detecting specific proteins contained in tumor-derived exosomes, we can confirm the origin of exosomes, diagnose related diseases, and evaluate the effect of treatment.

Based on the characteristics of the source cell, the exosomal lipid composition is diverse and generally contains cholesterol, sphingomyelin, phosphatidylserine, ceramide and et al. (Record et al., 2014; Leidal and Debnath, 2020). These lipids constitute a stable membrane structure of exosomes, which not only protect exosome contents from degradation but also regulate biogenesis of exosomes (Trajkovic et al., 2008; Skotland et al., 2017). In addition, as *in vivo* drug delivery has higher requirements for the stability of the carrier, the stable membrane structure of exosomes makes people pay more and more attention to the drug delivery of exosomes (EL Andaloussi et al., 2013; Phinney and Pittenger, 2017; Skotland et al., 2017). Kamerkar et al. assessed the effects of the exosomal drug carriers (carrying short interfering RNA specific to oncogenic KRAS) to uppress pancreatic cancer in multiple mouse models. They showed treatment with exosomal drug carriers efficiently suppressed cancer and observably enhanced overall survival as exosomes can protect themselves from phagocytosis by monocytes and macrophages (Kamerkar et al., 2017). In another study, they demonstrated the contribution of the exosomal lipids in the urine to investigate molecular markers of renal cell carcinoma patients (Del Boccio et al., 2012). Generally, the function of exosomal lipid needs more efforts to be considered effective for cancer diagnosis and prognostic potential.

Besides lipids and proteins, exosomes also contain multitudinous nucleic acids, which reflect the mutational status of the source cells. With the advances in next-generation sequencing approaches, these molecules were gradually recognized in exosomes, including almost all RNAs of the cell, ssDNA, and dsDNA (Valadi et al., 2007; Jiang et al., 2017). These nucleic acids in exosomes directly participate in transcription, translation, modification, and other processes to regulate gene expression and function of target cells (Jiang et al., 2017). For example, cells are able to selectively sort miRNAs into exosomes for gene regulation and intercellular communication by secreting to distant or nearby targets, revealing the potential functions and mechanisms of exosomal selective sorting in pathogenesis (Groot and Lee, 2020). In other study, Thakur and others first demonstrated that the majority of DNA of cancer-derived exosomes was double-stranded (Thakur et al., 2014). The evidence of nucleic acids further shows exosomes provide a crucial foundation in tumorigenesis factors and biomarkers in cancers.

## Common Exosomal Separation Techniques

Although exosomes play an irreplaceable role in early detection and treatment, they are small in size (30–150 nm), low in density (1.13–1.19 g/ml), and mixed with similar components (e.g., cell fragments, proteins) in the body fluids, which pose tremendous challenges for their separation (Cui et al., 2018; Lin et al., 2020). In addition, the biological activity of exosomes will be affected by different separation techniques (Paolini et al., 2016). To sum up, standardized separation and quantitative methods are pivotal to the study and clinical application of exosomes. For us, understanding the existing separation technology is the premise of developing more efficient and reasonable exosomes separation technology.

**FIGURE 4:**
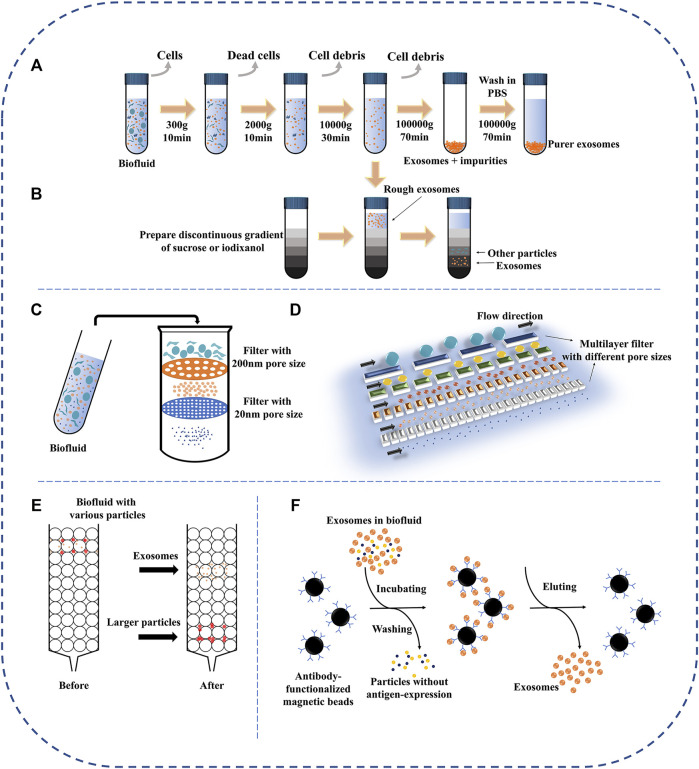
Schematic representation of common exosomal separation techniques. **(A)** Ultracentrifugation, **(B)** Density gradient centrifugation, **(C)** Dead-end filtration (DEF), **(D)** Tangential flow filtration (TFF), **(E)** Size-exclusion chromatography, and **(F)** Immunoaffinity.

Common separation methods mainly introduce ultracentrifugation, size exclusion chromatography, ultrafiltration, polymer precipitation, immunoaffinity (Figure 4). The merits and defects of these techniques are compared in Table 1.

### Ultracentrifugation

Ultracentrifugation is the most common technology at present, and about half of researchers use ultracentrifugation to separate exosomes (Zarovni et al., 2015). The principle of ultracentrifugation is based on differences in density and size between exosomes and impurities in the sample. The specific steps are shown in Figure 4A. First of all, the samples were centrifuged at 300 g, 2000 g, and 10,000 g, respectively. The larger cells, cell fragments, and dead cells could be removed (Théry et al., 2006). In some strategies, filtration can replace these low-speed centrifugal steps for the large-scale preparation of exosomes (Théry et al., 2001; Ji et al., 2008). The supernatant was then ultracentrifuged twice at higher speeds (>100,000 g) to yield exosomes. It should be noted that ultracentrifugation can only concentrate substances of similar density and size but not distinguish the exosomes carefully. As the most common method, ultracentrifugation has the advantages of mature technology, suitable for separating most samples, and low operating expenses. However, the entire separation process takes much time (>4 h), and the repeatability is poor/unstable. Secondly, despite several rounds of centrifugation, there are still many impurities in the precipitate (co-purifying protein aggregates, virion, Subcellular organelles), which may affect the results of subsequent mass spectrometry or protein quantitation (Tauro et al., 2012; Jeppesen et al., 2014; Zhang et al., 2018). Finally, high-speed centrifugation may cause damage to exosomes and reduce their biological activity (Jeppesen et al., 2014).

Density gradient centrifugation is an improved separation technology based on ultracentrifugation. The difference from ultracentrifugation is that density gradient centrifugation uses two or more separation media with different densities, such as sucrose and iodixanol (Tauro et al., 2012). The specific steps are also to remove large impurities by low-speed centrifugation, and then the sample is added to the top of the separation medium for ultracentrifugation (Figure 4B). The advantage of this method is higher isolation purity. However, it requires preliminary preparation, cumbersome operation, and longer centrifugation time (>16 h), which limits its clinical application (Gupta et al., 2018; Liu J. et al., 2021).

### Ultrafiltration

Ultrafiltration is a separation method based on molecular size and is one of the simplest methods for exosome separation. Exosomes are obtained by removing impurities through one or more filtering membranes with different pore sizes or the molecular weight cut off (MWCO). The pollutant larger than MWCO are quantitatively held back by the filtering membrane while other components (exosomes) smaller than the MWCO can pass through the filtering membrane structure along with the permeate (Li et al., 2017). Depending on the driving force, ultrafiltration can be classified as electric charge, centrifugation, and pressure.

For centrifugation-derive, He et al. established a highly efficient optimized ultrafiltration method. Relying on low-speed centrifugation, 0.22 μm filter and dialysis membrane with MWCO of 10,000 kDa, the device can remove impurities larger than 200 μm and the concentrate is reduced to 1/50 in comparison to the original volume (He et al., 2019). Compared to ultracentrifugation, the method requires only low-speed centrifugation and is suitable for large samples. However, it also has drawbacks, such as requiring additional commercial kits, being time-consuming (≈150 min), and not being suitable for blood.

For pressure-derive, it can be divided into dead-end filtration (DEF) and tangential flow filtration (TFF) (Figure 4C,D). DEF refers to the filtration form in which the liquid flow direction is the same as the filtration direction. Due to the rapid accumulation of the filter cake on the membrane surface, the permeation rate will continually reduce and eventually plug the filtering membrane (Busatto et al., 2018; Musumeci et al., 2018). Hence, DEF is best used to handle only small-scale liquids (e.g., syringe filter). In contrast to DEF, the flow direction is perpendicular to the direction of filtration in TFF. Because of this, the method can effectively avoid the formation of filter cake on the membrane surface, improving the utilization rate of the membrane and the stability of the equipment (Busatto et al., 2018). Kim et al. compared ultracentrifugation and TFF-based isolation (Kim et al., 2021). The results confirmed that the exosomal yield of the TFF-based isolation method increased by two orders of magnitude compared with that of ultracentrifugation.

For electric charge, Cho et al. developed an electro-migration method for attaining ultracentrifugation-level exosomal isolation from biological fluids. This method adopted the principle of TFF-based and electro-migration, used a 30 nm pore diameter filtering membrane for excluding impurities (e.g., protein) and preventing passage of exosomes (Cho et al., 2016). It proved that the exosomal recovery is 14 times higher than ultracentrifugation and the protein removal rate was 83.6% within 30 min. However, the complex structure of the device limits its clinical application.

In conclusion, ultrafiltration is one of the simplest methods for exosome separation. Exosomes can be separated from large volumes of liquid without expensive special equipment and harmful chemical reagents. Nevertheless, the clogging of exosomes on the surface of the filtering membrane may lead to a low recovery rate (Xu et al., 2018).

### Size-Exclusion Chromatography

Similar to the principle of ultrafiltration, SEC is a technology for separation based on the difference in molecular size. The specific step is that when the sample is added to the column containing porous beads (e.g., Sephadex, Sepharose, Sephacryl, BioGel P), the larger particles cannot enter the gel pores, and the elution speed is faster (Figure 4E) (Yang et al., 2020). On the contrary, small particles can enter the pores like a labyrinth, and the elution rate is slower to achieve the purpose of separation. Different from the powerful force of ultracentrifugation, exosome separation can be accomplished only by gravity or low-speed centrifugation in this method, maintaining the biological function of exosomes (Batrakova and Kim, 2015). Recently, several types of research have indicated that the method combining ultrafiltration and SEC can achieve a higher purity of exosomes (Nordin et al., 2015; Zeringer et al., 2015; Oeyen et al., 2018). Likewise, Guan et al. compared with the traditional ultracentrifugation and SEC (Guan et al., 2020). The separation results showed that more purified exosomes were isolated by SEC. For researchers focused on the biological function of exosomes and biomarkers, the purity of exosomes is a key indicator. Thus, it is a feasible strategy to conduct SEC after ultracentrifugation or ultrafiltration for them. In a word, SEC is a simple, prolongable, economical method for handing large-scale samples.

### Immunoaffinity

Immunoaffinity is a technique for separating and purifying biological particles based on the antigen-antibody specific reaction. The membrane of exosomes is rich in proteins and receptors, including ubiquitous common proteins and specific proteins. These ubiquitous proteins (such as tetraspanins, annexins) can bind to the corresponding antibodies to specifically isolate exosomes (Ruivo et al., 2017). In addition, it is possible to achieve efficient isolation of tumor-derived exosomes by targeting specific proteins contained in exosomes, such as EpCAM and antiA33 (Mathivanan et al., 2010b). Generally, antibodies need to be fixed on a certain carrier, such as magnetic particles, chromatography matrices, microfluidic devices (Figure 4F) (Zhang et al., 2018). At present, immunomagnetic beads are more common. The antibody-coated beads can specifically bind to the corresponding exosomes to distinguish them from the unbound impurities through the magnetic. Zarovni and others compared three methods for separating exosomes in plasma and cell supernatants (ultracentrifugation, density gradient centrifugation, and immunomagnetic beads), followed by verification with PCR and Elisa (Zarovni et al., 2015). The results show that the immunoaffinity method has more effective separation purity and separation advantages.

The immunoaffinity method has strong specificity, high isolation purity and can isolate a specific subclass of exosomes. However, since sufficient time is required for antigen-antibody binding, this method is time-consuming. In addition, there are disadvantages such as high separation cost and high requirements for reagent and storage conditions. This method is not an optimal choice for researchers who do not need high purity or specific subclasses of exosomes.

### Polymer Precipitation

The polymer precipitation method has been often used to isolate viruses or other biological macromolecules in the past (Zeringer et al., 2015). This method has become popular in exosomal isolation in recent years. The reagents used for polymer-based exosomes isolation mainly include protamine, acetate, protein organic solvent precipitation (PROSPR), polyethylene (PEG), among which PEG is the most common. (Brownlee et al., 2014; Deregibus et al., 2016; Gallart-Palau et al., 2016; Ryu et al., 2020). At the function of PEG, exosomal solubility is reduced to allow exosome precipitation. Then exosomes can be simply obtained by low-speed centrifugation. This method is simple to operate without complex devices or time-consuming procedures, and can handle large sample sizes, and is easy to combine with existing separation methods. Dash et al. focused on three exosome isolation methods, including PEG, PROSPR, and ultracentrifugation, indicating that the PEG-based approaches have high stability, well-dispersed, and good quality (Dash et al., 2021). Conversely, exosomes obtained by this method are susceptible to contamination with lipoproteins or virus particles, which may adversely affect subsequent analysis (e.g., proteomics, mass spectrometry). Recently, numerous researchers have combined this method with other separation methods to overcome the above shortcomings effectively (Ryu et al., 2020).

### Exosome Separation Kit

With exosomes gradually becoming the focus of research, various commercial kits have sprung up. At the same time, the application of the kit has become more and more extensive. Exosome’s isolation kit is not a specialized technology, and the principles have been described in detail above, such as chemical precipitation, immunoaffinity, size exclusion chromatography, centrifugation. The commercial products are easy to operate, does not require special equipment and are suitable for laboratories with insufficient equipment. At present, the common products mainly include Total Exosome Isolation kit (Invitrogen, United States) (TEI), Eloquence (System Biosciences, United States) (ExoQ), Exo-spin (Cell guidance systems, United Kingdom) (ExoS), *and so on*. These kits can isolate exosomes from various biological samples (e.g., serum, plasma, CSF), but the purity, quantity, and size distribution of the collected exosomes are significantly diverse (Helwa et al., 2017; Martins et al., 2018). For researchers concerned, it is crucial to choose a suitable product according to the purpose of the experiment. Similarly, the main defect of the kit is expensive and not suitable for mass sample processing.

### Realistic Large-Scale Production

In numerous pathological, biological and physiological researches, scientists have accomplished considerable achievements regarding the clinical application of exosomes for carriers of both genes and drugs. Despite many significant progresses have been made in the separation and purification of exosomes, it is a main challenge to develop larger-scale batch exosome production. This restricts the boundary of exosome-based biomedical treatment and researches. Hence, a reproducible, simple and good manufacturing practice (GMP)-compliant production platform is desired.

The procedures for large-scale production of exosomes are generally the use of multiple stacked cell culture flasks (e.g., T175 or T225), large fixed-bed bioreactors or stirred-tank bioreactors (Colao et al., 2018). The process contains complicated quality control, validation, quantification and characterization. Meanwhile, many issues need to be considered further, including shifting from small-scale laboratory isolation to large-scale commercial productions and moving from an experimental scheme to a commercial product (Whitford and Guterstam, 2019). In general, the key to large-scale production is the improvement of exosomal recovery rate and purity with high throughput and minimal cost. Lamparski et al. established a reproducible, rapid and credible method for the separation and production of clinical GMP exosomes (Lamparski et al., 2002). The procedure, which involved ultrafiltration, diafiltration and ultracentrifugation into 30% sucrose/deuterium oxide (D2O), achieved rapid isolation within 8 h, consistently exosome recovery of > 30%, high purity and cost-effectiveness.

The hollow-fiber bioreactor is an ideal method for the large-scale production. Cells are grown inside the fibers of the device, allowing media and metabolic waste to pass through but blocking larger secretions as exosomes. Watson et al. demonstrated the hollow-fiber system could increase exosome production by 5–10 folds (Watson et al., 2016). In general, the reactor can reduce contaminants and support high-density cell cultures.

Finally, further research and clinical application of therapeutic exosomes are inseparable from the large-scale GMP production of engineered exosomes. Therefore, further development of GMP protocols, more automated and digital production processes and strict quality control systems for engineered exosomes large-scale production are essential.

## Emerging Separation Technique

Although the above-mentioned traditional separation methods are the most widely used, there are also many disadvantages, such as large sample consumption, risk of damage to exosomes, low purity, and long time consuming, which are hard to meet the current increasing scientific research needs. With the development in recent years, several new separation technologies have been proposed and rapidly developed over the past decades. Among these new technologies, microfluidics offers an integrated platform and proves fascinating properties such as high purity, high throughput and low sample volumes. In addition, scientists have invented some techniques that use the properties of exosome phospholipid bilayers for separation and enrichment. The pivotal properties of these techniques are summarized in Table 2. By presenting and introducing these merits and defects of emerging strategies, we expect to propose future insights for next-generation device development.

### Membrane-Based Separation

Obviously, Exosomes are biological nanoparticles with lipid bilayer-closed structures (Brownlee et al., 2014). Thus, the surface of exosomes is rich in negatively charged phosphatidylserine, which makes a series of novel strategies possible (Deregibus et al., 2016).

**FIGURE 5:**
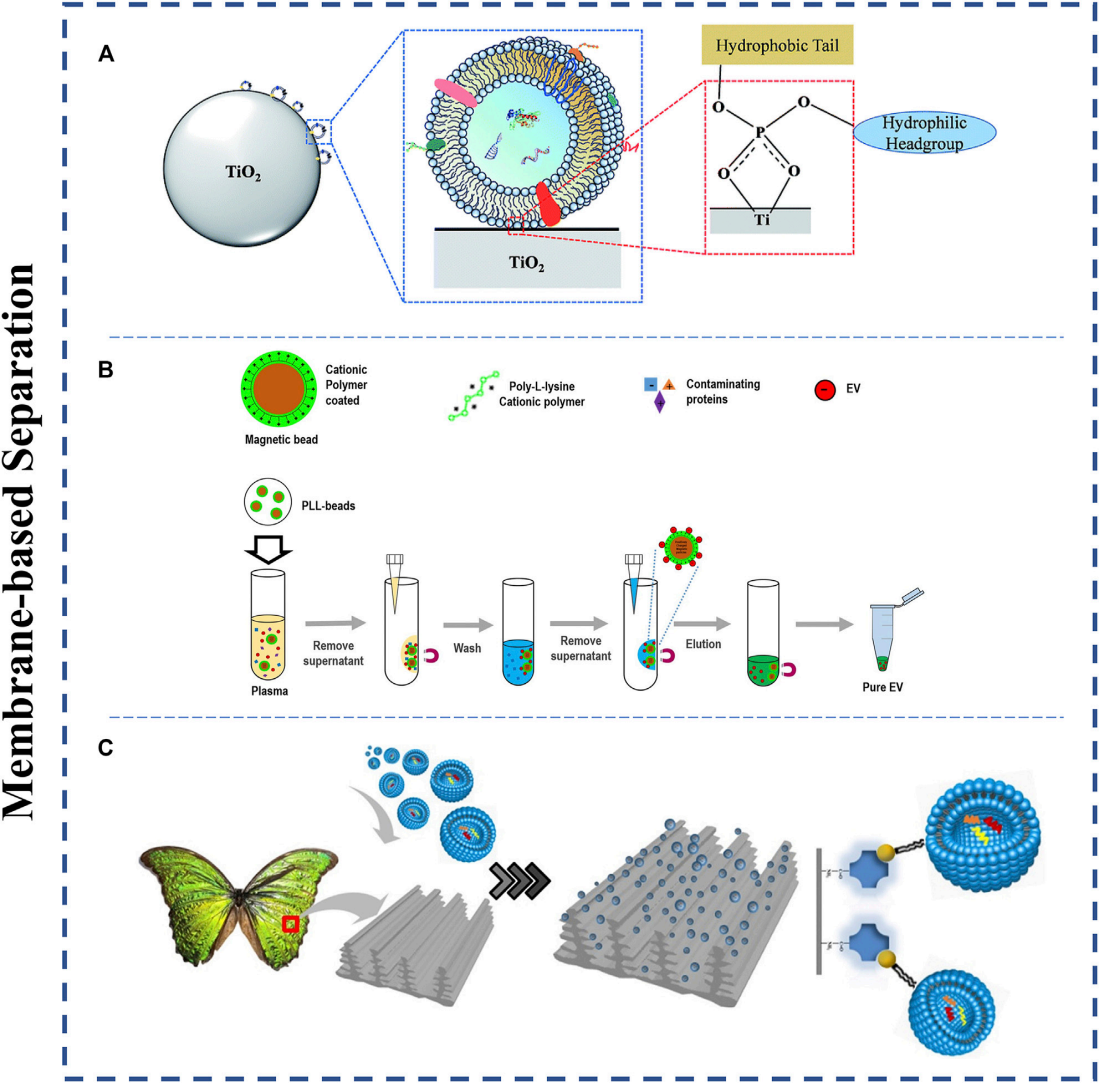
Schematic representation of Membrane-based exosome isolation techniques. **(A)** The phosphate groups on the membrane surface of exosomes can specifically bind to metal oxides (TiO_2_). Adapted from (Gao et al., 2019), copyright 2019 Royal Society of Chemistry. **(B)** The positively charged molecules enrich exosomes. Adapted from (Kim and Shin, 2021), copyright 2021 MDPI. **(C)** The lipid nanoprobes with lipid tail are capable of inserting into the exosomal membrane structure. The wings modified with lipid nanoprobes can promote the efficiency and speed of exosome binding to nanoprobes. Adapted from (Han et al., 2020), copyright 2020 Elsevier Ltd.

In exosomal systems, amphiphilic phospholipids constitute the main component of the exosomal lipid bilayer, forming the hydrophilic phosphate head located on the outer surface of the membrane (Gao et al., 2019). By this property, the phosphate groups can specifically bind to some metal oxides (e.g., TiO_2_, ZrO_2_). Based on this, Gao et al. performed exosome isolation by highly affinitive binding between micron-sized TiO_2_ particles and phosphate groups on the membrane surface of exosomes (Figure 5A) (Gao et al., 2019). By the TiO_2_-based isolation strategy, the method achieved an excellent separation efficiency of 93.4% within 5 min. Zhang et al. designed the magnetic TiO_2_ nanoparticles combining CD63 aptamer, which successfully isolated and captured 92.6% urinary exosomes within 10 min (Zhang et al., 2021).

Meanwhile, because of the negative charge with the exosomal lipid bilayer, some positively charged molecules can also be used to enrich exosomes easily. For instance, exosomes were successfully purified from human saliva, serum, and liver stem cells by positively charged protamine (Deregibus et al., 2016). Kim and shin produced an ion-exchange platform (ExoCAS-2) based on magnetic beads coated with a polycationic polymer for isolating exosomes with high purity and efficient yield from blood plasma (Figure 5B) (Kim and Shin, 2021). In brief, Poly-l-lysine (PLL) polymer-functionalized magnetic beads are mixed with the filtered plasma and incubated for a period of time. In the process, due to electrostatic reactions, PLL-coated beads with positive charges can easily combine with negatively charged exosomes. In the end, exosome-captured beads could be attracted by a magnet, and then the additional liquid was removed.

Additionally, Han et al. reported an innovative micro vortex chip by integrating butterfly wings functionalized with lipid nanoprobe into microfluidics for efficiently isolating exosomes from biological fluids (Figure 5C) (Han et al., 2020). In their study, due to the natural three-dimensional microstructure of the unique wings, it can provide a micro vortex when the liquid flows it, greater surface area and nanoprobe density. Meanwhile, the lipid nanoprobes labeled to the wings can insert into exosomes quickly to capture it. Based on the described properties of the microfluidic chip, it effectively promoted microscale mass transfer of nanoparticles and thus achieved about 70% separation efficiency of exosomes.

Membrane-based separation methods have shown a powerful ability of isolating greater exosomes. Meanwhile, the methods hardly rely on the surface proteins of exosomes that can avoid low purity or yield caused by inherent heterogeneity of exosomes. It is worth noting that the isolated exosomes may contain other impurities with membrane because the methods are mainly based on lipid bilayer-closed structures.

### Microfluidics

Microfluidics has been deemed a promising method that can integrate sample processing, analysis, detection, and other processes into a chip, thus realizing miniaturization, integration, high-throughput capacity, and low-time consumption (Jiang et al., 2014). At present, on account of these advantages, microfluidic chips are gradually being used as a powerful tool for conveniently, efficiently isolating exosomes.

Microfluidics can be combined with common separation techniques for exosomes separation via exosomal physical and chemical features (e.g., surface antigens, density, size). On the other hand, Microfluidics devices in combination with external forces are also emerging as efficient platforms (e.g., acoustic field, magnetic field, or electric field). Here, we introduced the different microfluidics-based techniques for exosomes isolation roundly.

#### Physical Property-Based Microfluidics

The physical properties of exosomes have been discussed in detail. Current, physical-property-based microfluidic isolation strategies have been emerging as powerful methods since the method can achieve label-free isolation and exosomal uniformity. Physical-property-based microfluidic devices usually contain nanoporous membranes, nanofilters, microvillus, acoustic field, and electric field to filter or trap exosomes. Here, based on the presence of external forces, we divide physical property-based microfluidics into two categories: active and passive isolation methods.

##### Active Isolation Methods

Scholars have integrated microfluidic systems with various external forces to achieve faster, higher and normative strategies of exosomal isolation. The active isolation methods included electrical, centrifugal, acoustical forces.

**FIGURE 6:**
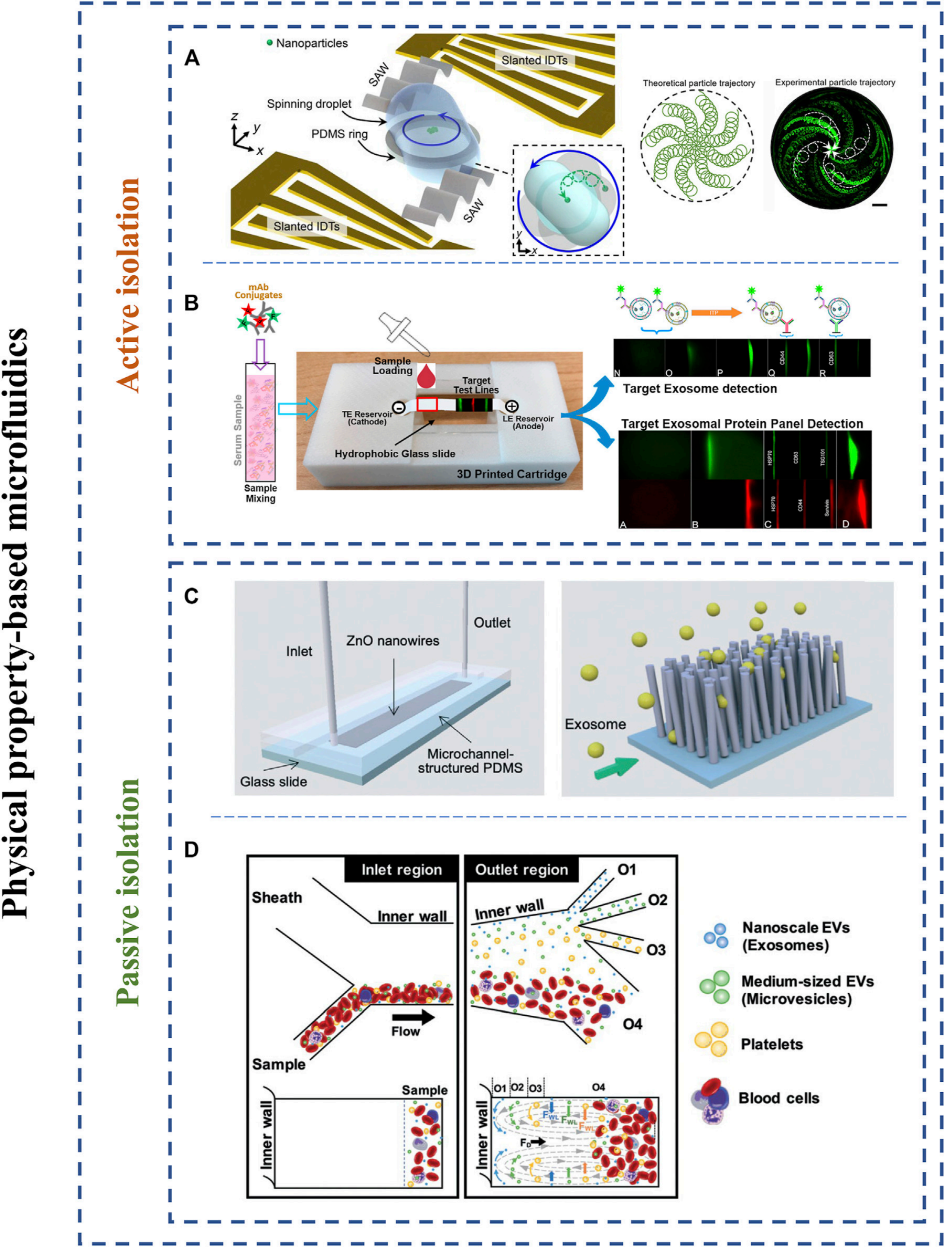
Schematic representation of physical property-based microfluidic isolation techniques. **(A)** An acoustic-based separation microfluidic chip employing acoustic forces and droplet spinning for isolation of exosomes from biofluids. Adapted from (Gu et al., 2021), copyright 2021 American Association for the Advancement of Science. **(B)** An electrical-based separation device integrated the focusing power of isotachophoresis and paper-based filtering ability. Adapted from (Guo et al., 2020), copyright 2020 Elsevier Ltd. **(C)** A ZnO nanowires array for exosome capture. Adapted from (Suwatthanarak et al., 2021), copyright 2021 Royal Society of Chemistry. **(D)** Hydrodynamic-based microfluidic strategy for isolating exosomes from whole blood. Adapted from (Tay et al., 2021), copyright 2021 Royal Society of Chemistry.

In the acoustical fields, different particle sizes are a pivotal element. The larger particles will be subjected to greater acoustic forces, hence separating various particles. In the past, acoustic-based methods have been successfully implemented cells and single microparticles separation (Ding et al., 2012). Recently, Wu and others fabricated an acoustic-based microfluidic device consisting of two acoustofluidic modules for removing larger blood components and separating exosomes, respectively (Wu et al., 2017). The capture results indicated that the separation efficiency of exosomes was achieved 98.4%. Zhao et al. demonstrated a disposable acoustofluidic chip with unidirectional interdigital transducers that can realize versatility and biocompatibility (Zhao et al., 2020). As another strategy, Gu et al. reported an acoustofluidic technique employing acoustic streaming and droplet spinning that can identify and separate exosome subpopulations (Figure 6A) (Gu et al., 2021). With regard to this acoustofluidic centrifuge, particles in a droplet were pushed toward the center of the droplet under the action of acoustic radiation forces and streaming generated by high-frequency acoustic waves. The device can achieve high separation efficiency (80–86%) and extremely short processing time (≤1 min). Unfortunately, the study needs to be improved the low sample volume per assay and potential evaporation issue.

Electrical-based separation mainly relies on electric field intensity, diameters, and electrical properties of the particles (Cheng et al., 2015). Cho et al. demonstrated an electrical-based isolation system possessing an electric field across the commercial dialysis membrane (30 nm pore size) for isolating exosomes and excluding nano-sized impurities (Cho et al., 2016). This study achieved 65% exosomes recovery by quantifying RNA amount. Meanwhile, the Ayala-Mar group developed the direct current-insulator-based dielectrophoretic microfluidic chip that can isolate and discriminate exosome subpopulations (Ayala-Mar et al., 2019). Guo et al. proposed a paper-based isotachophoresis device that was capable of rapid separation and detection of exosomes derived from cancer cells/tissues (Figure 6B) (Guo et al., 2020). The method integrated the focusing power of isotachophoresis and paper-based filtering ability. Compared with traditional enhanced Elisa, the detection limit of the device is increased by 30-fold. With future improvement on separation purity and large-scale clinical studies, electrical-based methods could be a promising strategy for on-chip rapid separation and diagnostics.

Centrifugal microfluidics was achieved to capture and isolate bioparticles in one single platform. Recently, Woo et al. developed a procedure consisting of two filtration chambers (pore diameter = 600 and 20 nm, respectively) for automatically enriching and separating exosomes and other vesicles from biological samples (Woo et al., 2017). The centrifugal platform required only low g-force (< 500 g) instead of 150,000 g of UC, quickly completed processes of enrichment (< 30 min) and provided a high recovery (>95% for urinary EVs). In addition to the outstanding capability, simple operation and relatively low cost made the device a promising strategy for both clinical research and the laboratory.

Overall, the active isolation methods can realize continuous-flow, biocompatible and label-free exosome separation to research the role of exosomes for cancer diagnostics.

##### Passive Isolation Methods

Researchers have also incorporated strategies based on complicated channel structures or hydrodynamic characteristics into microfluidic devices. Passive isolation methods generally included nanoporous membranes, nanofilters, nanopillar arrays, and hydrodynamic characteristics.

For example, Suwatthanarak et al. developed the ZnO nanowire array modified exosome-targeting peptides for efficient exosomes enrichment from cultured medium of human breast cancer cells (Figure 6C) (Suwatthanarak et al., 2021). Meanwhile, the captured exosomes by the ZnO nanowire array were capable of releasing under a neutral salt condition that could protect the collected exosomes and not affect their downstream applications. The peptide-nanowire platform reached the exosome recovery of 70% approximately from 1 ml suspension of exosomes in 20 min collecting time. In the future, clinical samples or cancer-derived exosomes spiked in serum should be used to further confirm the performance of this device.

Pillar-based microfluidics (Deterministic Lateral Displacement, DLD) consists of one or more arrays of optimal pitch, gap, and diameter of pillars for various particles isolation (Lin et al., 2020). The principle of the method is that particles larger than the critical diameter are capable of migrating at a certain angle defined by the pillar spacing and array gradient (Iliescu et al., 2019). For instance, Wunsch and groups first produced nanoscale DLD arrays consisting of consistent gap sizes ranging from 25 to 235 nm (Wunsch et al., 2016). The method demonstrated the nanoparticles with diameters from 20 to 110 nm could successfully isolate from biological samples with sharp resolution, presenting hopeful potentials as chip-based liquid biopsies in monitoring early cancer screening and diagnostics.

Hydrodynamic-based microfluidic strategies (e.g., viscoelastic flow or inertial flow) have also indicated that this principle can effectively separate various particles from complicated biofluids. Compared to other methods, hydrodynamic-based microfluidic devices can be implemented without complicated channel structures or additional external force fields. This can simplify the fabrication process and operation difficulty of microfluidic devices. To illustrate, Tay and others have developed a novel inertial-based microfluidic device for directly separating exosomes and microvesicles from whole blood with 15% (±3.8%) separation efficiency within an hour (Figure 6D) (Tay et al., 2021). Although the separation efficiency is limited, the method does not require functionalization with antibodies or external force field and performs directly separation of whole blood, hence providing a low device cost, portable, and promising strategy.

In summary, due to taking advantage of label-free, portability, and reproducibility, various passive isolation methods will spring up like fountains.

#### Immunoaffinity-Based Microfluidics

The immunoaffinity-based microfluidic devices depend on the antigen-antibody reaction for isolating specific exosomes. Hence, the critical element for efficiently isolating exosomes is the selection of suitable antibodies, promoting microscale mass transfer, increasing particles-surface collisions (or increasing contact surface area) (Zhang et al., 2019; Li et al., 2020). In most cases, immunoaffinity capture can be achieved by stationary antibody-coated mediums and mobile antibody-coated mediums.

##### Mobile-Coated Mediums

**FIGURE 7:**
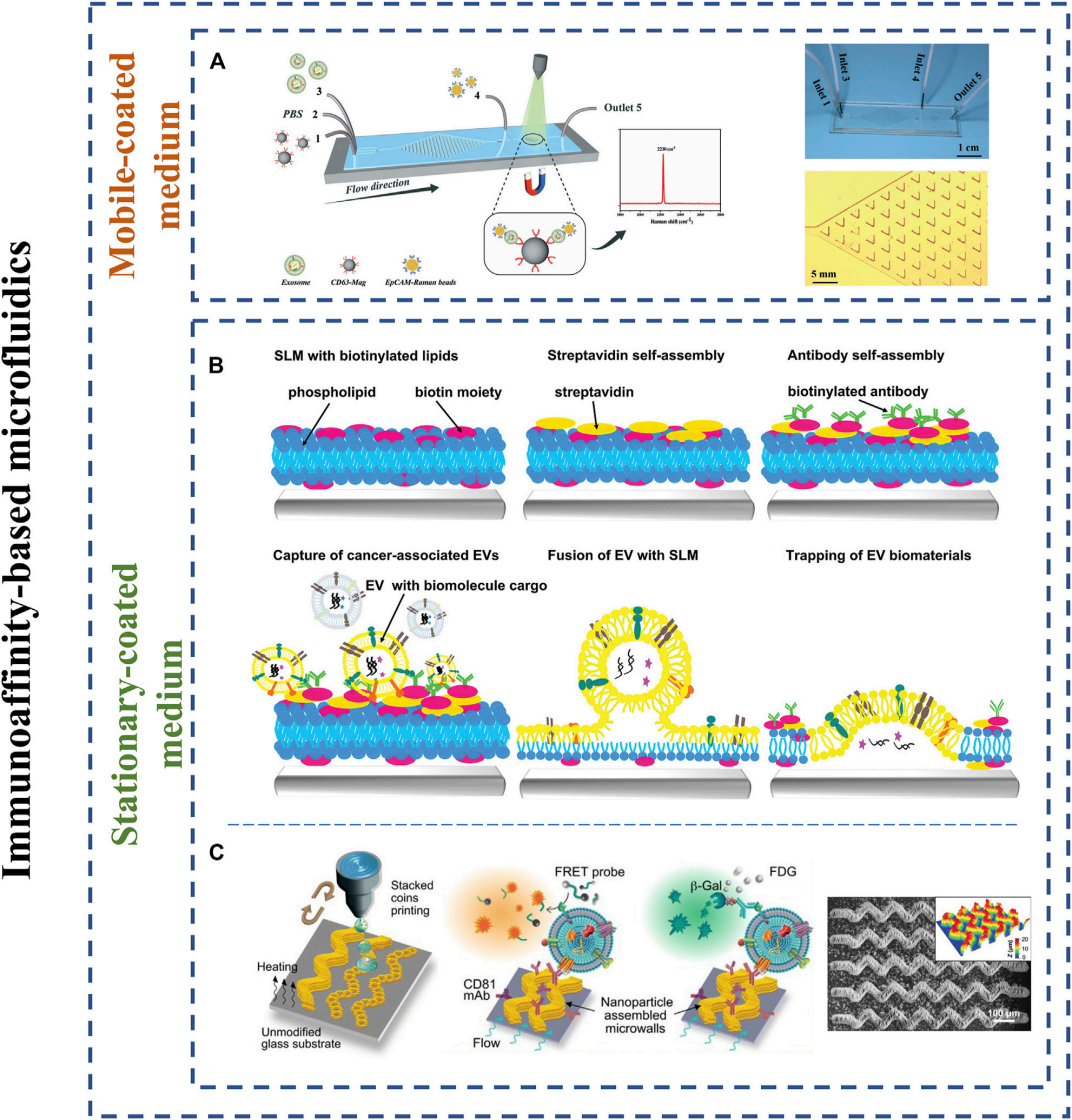
Scheme of immunoaffinity-based microfluidics for exosome isolation and enrichment. **(A)** Microfluidic Raman chip for exosome isolation and detection. Adapted from (Wang et al., 2020), copyright 2020 Royal Society of Chemistry. **(B)** Scheme of lipid membranes microarrays functionalized with antibodies. Adapted from (Liu H. Y. et al., 2021), copyright 2021 Wiley-VCH Verlag GmbH & Co. **(C)** 3D nanopatterned EV-CLUE chip were manufactured by colloidal inkjet printing. Adapted from (Zhang et al., 2020), copyright 2020 American Association for the Advancement of Science.

Mobile coated mediums refer to magnetic beads or other magnetic nanomaterials here. These antibody-coated nanoparticles inherently provide a larger surface area and flexibility in handing which enhance the capture and release efficiency of exosomes from microfluidics to perform better downstream analysis. For instance, Liu et al. modified the surface of nanoparticles consisting of silica shell and Fe_3_0_4_ core with two conjugated antibodies (anti-CD63 and anti-myosin light chain) to capture CD63-expressing exosomes and target injured cardiomyocytes, respectively (Liu et al., 2020). Sancho-Albero et al. developed magnetic beads microfluidic platform functionalized with anti-CD9 antibodies capable of isolating magnetic bead-captured exosomes from whole blood by NdFeB permanent magnets (Sancho-Albero et al., 2020). However, one apparent defect of the platform was unable to realize the integration of isolation, purification and detection onto the same chip, which increased additional operations and the probability of sample contamination. Recently, Wang et al. reported an integrated microfluidic Raman biochip capable of isolating and detecting serum exosomes on the same chip where anti-CD63 conjugated magnetic beads and EpCAM-functionalized Raman beads were utilized to distinguish between healthy patients and prostate cancer patients within an hour (Figure 7A) (Wang et al., 2020). In the approach, anti-CD63 antibody-labeled magnetic beads were first used to isolate CD63-expressing exosome through mixing region consisting of a staggered triangular micropillar array, followed by magnetically fixing on detection chamber and detecting these captured exosomes by Raman beads functionalized with anti-EpCAM antibody. Finally, further SERS analyses were introduced to detect clinical serum samples. The device could entirely handle and detect 20 μl clinical samples in <1h at an isolation efficiency of 72.5%. However, the absence of more clinical sample data in the research could serve as starting points of future development.

In brief, mobile antibody-coated methods have the advantages of high specificity, throughput, and capture efficiency. However, for large volumes of samples, the method is not optimal and has a high experimental cost.

##### Stationary-Coated Mediums

Stationary-coated mediums mainly rely on interactions between exosomes and antibodies/aptamers immobilized on the surface of microchannels for affinity capture. It was recently reported that the limitations of mass transfer and hydrodynamic resistance because of the greatly low diffusion of bioparticles in microchannels restrict exosomes to contact with antibodies/aptamers modified on the channels, which extremely reduces binding efficiency (Zhang et al., 2019; Li et al., 2020). Thus, effective promotion of mass transfer of bioparticles, increase of surface area and probe density are essential.

Kang et al. developed a device of joint isolation and release of desired exosomes providing exosomal recovery yield of above 76% (Kang Y.-T. et al., 2020). Again, Kang and the group proposed a dual-utilization chip functionalized with anti-MCAM and anti-MCSP antibodies for simultaneously isolating cancer-exosomes and circulating tumor cells in a single platform (Kang Y. T. et al., 2020). It is worth noting that the method first integrated isolation of cancer-exosomes and circulating tumor cells from single samples in a single microarray. The ability of dual isolation and molecular detection will allow for further identify and enhance cancer detection and clinical applications in the future.

Liu et al. established supported lipid membranes microarrays functionalized with antibodies for recognizing and capturing cancer-specific exosomes (Figure 7B) (Liu H. Y. et al., 2021). In this research, the lipid microarray was first structured using lipid dip-pen nanolithography, followed by incubating and self-assembling onto the lipid microarrays by using Biotin-PE, streptavidin, and specific biotinylated antibodies in a prearranged sequence. Finally, the experiment results show that the platform can detect and capture cancer-associated exosomes from unpurified or purified 30–50 μl samples volumes within 2 hours. While several clinical tests on pancreatic cancer were accomplished to identify diagnostic potential of CD63 and EpCAM, the ability can be further improved due to few clinical samples.

To further improve the capture efficiency, various micromixing approaches have been proposed to enhance microscale mass transfer to increase exosomes-antibodies collisions (e.g., herringbone mixers). For instance, employing engineered colloidal self-assembly (CSA), Zhang et al. developed a microfluidic platform (Nano-HB) consisting of self-assembled three-dimensional herringbone nanopatterns functionalized with specific antibodies (Zhang et al., 2019). The platform promoted the efficiency of exosome binding to antibodies and reduced near-surface hydrodynamic resistance. Thus, low levels of tumor-associated exosomes in plasma can be detected at concentrations as low as ten exosomes μL^−1^. The method provided an advantageous platform for detecting diagnostic markers of non-invasive screening with high sensitivity and specificity. However, the CSA-base strategy depends on supererogatory surface pretreatment and complex production process that may limit large-scale clinical studies. To address these issues, Zhang and the group reported again EV-CLUE chips (three-dimensional nanopatterned microchips) that were manufactured by high-resolution colloidal inkjet printing method (Figure 7C) (Zhang et al., 2020). Based on the printing method, the microchip consisted of continuous 3D sinusoidal patterns functionalized with specific antibodies that can increase surface area, enhance microscale mass transfer and allow the drainage of the boundary fluid. Compared to the previous Nano-HB, the EV-CLUE chip could achieve on the unmodified glass surface, enhancing the repeatability and facilitating the success ratio of largescale fabrication. Finally, the results showed that the EV-CLUE chip possessed 78.2 ± 2.6% efficiency for SKOV3. While the demonstrated principle and capture efficiency of EV-CLUE chips resembled previously mentioned Nano-HB platform, the chip is capable of more efficient detection for isolated exosomes and more flexible fluid control due to the use of pneumatic pumps. The device provided a beneficial liquid biopsy platform to further cancer diagnosis and treatment.

## Conclusion

Due to their clinical potential and unique biological functions, exosome researches and applications have brought substantive breakthroughs in drug delivery, non-invasive disease diagnosis, treatment and other fields. In the last few decades, a major limit factor in clinical applications, the drawbacks of common exosomal isolation methods, has inspired efforts for developing emerging separation platforms. At first, researchers drew inspirations from individual or combinations of various conventional schemes that previously were used to separate other larger particles (e.g., CTCs) from bio-fluids. Up to now, incorporating emerging technologies (e.g., microfluidics, electrical, centrifugal, acoustical forces) into exosome isolation technologies has widely developed and become more consummate than before. Microfluidic devices for exosome isolation and purification will be expected to be promising tools for early detection and biomedical applications. Despite the great progress made, it is clear that current separation methods are not perfect. In addition to achieving high-purity exosome isolation, more integrated, high-throughput, high-recovery-rate devices will break a promising avenue for exosome-based diagnostics and biomedical applications in the years ahead.

In this article, we summarized the merits and shortcoming of the latest advances for exosomal isolation and purification, including common and emerging strategies. Additionally, the biogenesis, contents of exosomes and their central functions as tumorigenesis factors and biomarkers in cancers were discussed in detail. We also reviewed the current challenges and future directions in the field. The aforementioned challenges, when addressed, exosomes will provide a powerful platform for cancer detection and treatment.
